# Prognosis and Influencing Factors of Early Microsurgery for Severe Hypertensive Brainstem Hemorrhage

**DOI:** 10.1155/2022/5062591

**Published:** 2022-09-22

**Authors:** Xianbing Meng, Qian Wang, Xianguang Pei, Fangmin Xie

**Affiliations:** ^1^The Second Affiliated Hospital of Shandong First Medical University, Taian, Shandong Province 271000, China; ^2^The First Hospital of Handan City, Handan, Hebei Province 056002, China

## Abstract

**Objective:**

To investigate the prognosis and influencing factors of early microsurgery for severe hypertensive brainstem hemorrhage.

**Methods:**

The clinical data of 19 patients with severe hypertensive brainstem hemorrhage treated in the Department of Neurosurgery of the Second Affiliated Hospital of Shandong First Medical University between January 2018 and December 2021 were retrospectively analyzed. The clinical efficacy and risk factors affecting the prognosis were analyzed by chi-square test and multivariate logistic regression.

**Results:**

A total of 19 patients with severe hypertensive brainstem hemorrhage were treated by early microsurgery, including 14 cases by subtemporal approach and 5 cases by retrosigmoid approach. After 3 months of follow-up, 6 patients died and 13 patients survived. The 30-day and 90-day mortality rates were 21.1% and 31.6%, respectively, and the good prognosis rate was 15.4%. Univariate analysis showed that hematoma volume and hematoma clearance rate might be the factors affecting the prognosis of patients with severe hypertensive brainstem hemorrhage; the observed difference was statistically significant (*P* < 0.05). Multivariate logistic regression analysis further confirmed that hematoma volume was an independent factor affecting the death of patients with brainstem hemorrhage (*P* < 0.05), while hematoma volume (*B*: 2.909, OR: 18.332, 95% CI: 1.020–329.458, *P*: 0.048) was a risk factor.

**Conclusion:**

Hematoma volume resulted as an independent factor affecting the death of patients with severe hypertensive brainstem hemorrhage. Early microsurgical clearance of brainstem hematoma contributed to reducing the 30-day and 90-day mortality and improving the prognosis of patients.

## 1. Introduction

Hypertensive brainstem hemorrhage (HBSH), which is characterized by acute onset, rapid progress, high mortality, and disability rate, is one of the most dangerous types of hemorrhagic stroke [1, 2]. According to previous studies, its annual incidence is about 2–4/100,000 [3, 4], and it mostly affects patients 40 to 60 years old, being more common in men than in women [2]. A hematoma usually forms rapidly after brainstem hemorrhage, leading to coma, central fever, tetraplegia, respiratory and circulatory failure, and other symptoms [2, 5, 6]. Conservative treatment can be used for patients with a small amount of brainstem hemorrhage and clear consciousness [7]; However, for patients with severe hypertensive brainstem hemorrhage (SHBSH) (bleeding volume >5 mL, GCS score <8 points), conservative treatment cannot effectively prevent the progress of the disease, and the mortality is as high as 80–100% [8]. Scholars in China and abroad have been working meticulously to improve the treatment of brainstem hemorrhage, reduce the mortality and disability rate, and improve the prognosis of patients.

Due to its complex anatomy, an important function, and high operation risk, the brainstem has long been regarded as the “forbidden area of neurosurgery” [9]. With the in-depth study of the anatomical structure and physiological function of the brainstem, it was confirmed that there is a relatively “safe operation area” in the brainstem [10–12]. Microsurgery is expected to become one of the most important ways to improve the prognosis of SHBSH patients [2, 11]. Nevertheless, whether surgical treatment could improve the prognosis of patients with brainstem hemorrhage still remains controversial [13, 14]. Rohde et al. [7] and Haines and Mollman [15] argued that patients with brainstem hemorrhage accompanied by instant coma and severe neurological deficit had a low probability of survival and were not suitable for surgical treatment. The United States and European Guidelines for Spontaneous Intracerebral Hemorrhage also do not recommend surgical treatment for patients with HBSH [16, 17]. Recent studies have reported that some patients with HBSH had their symptoms improved after microsurgical treatment [3, 4, 18, 19]. However, there are few reports on the microsurgical treatment of SHBSH, and its safety and effectiveness have not been fully elucidated. In this study, we retrospectively analyzed the data of 19 patients with SHBSH treated by microsurgery in the early stage and further discussed its clinical efficacy and risk factors affecting the prognosis of patients.

## 2. Materials and Methods

### 2.1. General Information

A total of 19 patients with SHBSH treated by microsurgery in the Department of Neurosurgery of the Second Affiliated Hospital of Shandong First Medical University from January 2018 to December 2021 were included in the study. There were 13 males and 6 females, with the age range from 32 to 64 years old and an average of 49.1 years old. Glasgow Coma Scale (GCS) showed 17 cases with 3–5 points and 2 cases with 6–8 points.

This study was approved by the ethics committee of the Second Affiliated Hospital of Shandong First Medical University, and the patients' families signed the informed consent.

### 2.2. Inclusion and Exclusion Criteria

Inclusion criteria were the following: (1) brainstem hematoma volume >5 ml confirmed by brain CT; (2) GCS score ≤8 points, with/without progressive aggravation of consciousness; (3) onset time ≤24 hours and stable circulation and respiration; and (4) complete clinical case data.

Exclusion criteria were (1) secondary brainstem hemorrhage caused by vascular malformation, cavernous hemangioma, metastasis, etc.; (2) GCS score of 3 points and unstable vital signs; and (3) complicated with important organ failure or coagulation disorders.

### 2.3. Imaging Examination

Brainstem hemorrhage was confirmed by brain computed tomography (CT) examination in all of the 19 patients, and the hemorrhage location was limited to the pons. According to the Tada formula [20], the amount of hemorrhage was calculated. The hematoma amount was 5–10 ml in 12 cases and ≥10 ml in 7 cases. Among them, 2 patients were complicated with acute obstructive hydrocephalus.

### 2.4. Surgical Analysis

All of the 19 patients were operated within 24 hours after onset. Before the operation, the location of the hematoma was determined based on brain CT scanning and MPR (multiplanar reformatting), and the individualized surgical approach was designed according to the anatomical location and expansion direction of the hematoma. Among these patients, 14 were managed by a subtemporal approach. During the operation, the tentorium cerebelli was incised to expose the lateral brainstem operation area, and the location of the brainstem hematoma was determined according to the condition. Next, the brainstem was incised longitudinally, and the hematoma clearance operation strictly limited in the hematoma cavity was performed under the microscope. The operation did not exceed the boundary of the hematoma in order to avoid brainstem injury as much as possible. In 5 cases with retrosigmoid approach, the location of the hematoma was identified under a microscope, and the lateral pontine was incised under direct vision to clear the brainstem hematoma. In case of active hemorrhage, low-power bipolar electrocoagulation was used to stop bleeding. When patients were combined with acute obstructive hydrocephalus, the lateral ventricle was drained first.

### 2.5. Efficacy Evaluation

The rehemorrhage rate, infection rate, and 30-day and 90-day survival rate of patients were analyzed after the operation. The patients were followed up for 1 and 3 months, respectively. The survival of patients was investigated, and the prognosis of surviving patients was evaluated by a modified Rankin scale (mRS) [21] (Table 1), where mRS ≤3 points indicated good prognosis and mRS with 4–6 points indicated poor prognosis.

### 2.6. Statistical Analysis

Statistical analyses were performed by SPSS software (version 25.0). The enumeration data were represented as a number of cases or rates. The chi-square test of the cross table was used to compare the differences in univariate analysis, after which the factors with statistical significance in the chi-square test were further analyzed with ordinal logistic regression. A *P* value <0.05 was considered to be statistically significant.

## 3. Results

### 3.1. The 30-Day and 90-Day Mortality in Patients with Brainstem Hemorrhage

Among the 19 patients with severe hypertensive brainstem hemorrhage treated by microsurgery, 6 died and 13 survived. The 30-day and 90-day mortality rates were 21.1% and 31.6%, respectively (Table 2). The mRS score of 13 surviving patients was 4.08 ± 0.64 at 3 months of follow-up and 4.09 ± 0.70 at 6 months of follow-up. Among surviving patients, 2 had mRS score ≤3 points, and the good prognosis rate accounted for 15.4% of the total number.

### 3.2. Univariate Analysis of Prognosis of Patients with Brainstem Hemorrhage

Univariate analysis (Table 3) showed that the effects of factors including GCS score, onset-operation time, complicated with hydrocephalus, gender, age, and hematoma type had no significant difference on prognosis (all *P* > 0.05). However, the differences in the influence of hematoma volume and hematoma clearance rate on prognosis showed statistical significance (*P* < 0.05), suggesting that these factors might affect the prognosis of brainstem hemorrhage.

### 3.3. Multivariate Logistic Regression Analysis for the Prognosis of Patients with Brainstem Hemorrhage

Taking death (death/survival: 1/0) as the dependent variable and the hematoma volume (>10 ml/5–10 ml: 1/0) and hematoma clearance rate (≥80%/<80%: 1/0) as the independent variables for multivariate logistic regression analysis with the input method revealed that the independent factor affecting the death of patients with brainstem hemorrhage was the hematoma volume (*P* < 0.05), while the hematoma volume (*B*: 2.909, OR: 18.332, 95% CI: 1.020–329.458, *P*: 0.048) was also a risk factor (Table 4).

### 3.4. Typical Cases of Patients with Severe Brainstem Hemorrhage (Figure 1)

## 4. Discussion

HBSH accounts for 6–10% of all hypertensive intracerebral hemorrhages [14], with pons hemorrhage being the most common [22]. Hemorrhage mainly originates from the perforating artery of the paramedian branches supplying the brainstem [3]. Hypertension and atherosclerosis are the most common causes of brainstem hemorrhage [2, 9]. Previous studies suggested that the following are mechanisms of pathological injury caused by brainstem hemorrhage: First, the mechanical destruction of hematoma directly leads to the primary injury of the brainstem; second, the compression of hematoma leads to the secondary injury of the brainstem such as local tissue ischemia, edema, or inflammatory reaction caused by hematoma catabolic products [9]. It was previously confirmed that the necrosis of brain tissue around the hematoma begins within 6 hours of the onset of intracerebral hemorrhage, increases after 12 hours, and reaches the peak within 24 hours [23]. Therefore, the prompt clearance of the brainstem hematoma after the onset of SHBSH is essential for reducing the primary injury and preventing secondary injury, which is conducive to the protection of brainstem nerve function [9, 23].

Hematoma clearance by craniotomy and stereotaxis and/or navigation-guided hematoma puncture and drainage are the main surgical methods for treating PBSH [2, 9]. Early studies have reported that stereotaxis or navigation-guided puncture of brainstem hematoma could benefit some patients, but hematoma cannot be completely cleared with such an approach, and once the bleeding starts, it cannot be stopped under direct vision, thus limiting its application [24, 25]. Hematoma clearance by craniotomy is a classic surgical method of PBSH, which can be performed under direct vision, with accurate hemostatic effect and decompression at the same time [2]. The optimal approach is essential for successful brainstem hemorrhage surgery [2]. Due to the complex anatomical structure of the brainstem, the principle of the shortest approach and least injury must be followed when planning the surgical path so as to avoid iatrogenic injury [26]. In this study, we followed Brown's Rule [27], i.e., multiplanar reformatting of brain CT scanning was combined, and individualized surgical approaches were adopted. There were 14 cases of hematoma cleared by subtemporal approach and 5 cases by retrosigmoid approach. If it is suspected that the hematoma is located in the middle and upper ventrolateral part of the pons, the subtemporal approach can be adopted. During the operation, the temporal lobe was gently lifted to expose the superior petrosal sinus, and the tentorial edge of the cerebellum was cut so that the middle and upper sides of the pons could be reached. Through this area, it was possible to reach the focus quickly, and it was safer to cut the brainstem, which was consistent with the previous literature [19]. For patients with ventrolateral pontine hematoma, the retrosigmoid approach could be adopted, by which the supratrigeminal and lateral pontine safety entry zones could be reached [28]. The difficulty of hematoma clearance by craniotomy is the localization of brainstem hematoma during operation. If the hematoma breaks through the surface of the brainstem, a hematoma can be easily located and identified, and the cavity could be accessed through the break [9]. If the hematoma is close to the surface of the brainstem, color changes or local uplift occurs, and the hematoma cavity can be accessed by cutting the brainstem longitudinally in the local lesion [9, 19]. If the hematoma is confined to the brainstem tissue and there is no change on the surface, the hematoma cavity can be accessed through the safe entry zone [11, 12]. In addition, during the hematoma clearance, the brainstem supplying arteries and draining veins should be avoided as much as possible so as to avoid iatrogenic injury.

Whether microsurgery can improve the prognosis of SHBSH patients remains controversial. In their study, Lan et al. found that the mortality of SHBSH patients in the conservative treatment group was 70.42%, while that in the craniotomy group was 30.43% [8]. The mortality in this study was 31.6% (6/19), which was consistent with the previous literature report. Jang et al. reported that the 30-day mortality of patients with brainstem hemorrhage was 39.1%, the 90-day mortality was 49.8%, and the 90-day good function recovery rate was 9.6% [6]. The 30-day and 90-day mortality rates in this study were 21.1% and 31.6%, respectively, and the good prognosis rate was 15.4% (2/19), which was better than the survival rate reported in the previous literature [6, 8].

Although previous studies argued that brainstem hemorrhage requires early surgery, there is no unified standard in China and abroad for the best timing of HBSH surgery. Lan et al. [8] found that the nervous system recovery in the early operation group (≤ 6 h) of PBSH patients was better than that in the late operation group (> 6 h), so they suggested that the best operation timing might be to receive surgical treatment within 6 hours after the onset of the disease. In their study, Chen et al. [3] retrospectively analyzed the data of 52 patients with brainstem hemorrhage and found that 12–48 hours after the onset of PBSH was the best timing for surgery. The consensus of Chinese experts recommends 6–24 h after hemorrhage as the best timing for surgery [9]. Some studies suggested that the surgery timing was an important factor affecting the prognosis of brainstem hemorrhage. Yet, our results revealed that the operation timing was not statistically significant (*P* > 0.05), which was inconsistent with the previous studies, and may be related to the small sample size [8].

At present, the prognosis and influencing factors of hypertensive supratentorial hemorrhage have been well studied [16, 17]. However, brainstem hemorrhage-related studies are inadequate. Raison et al. believe that the prognosis of SHBSH patients depends on the location of hematoma, extent of hemorrhage, and the condition at the time of onset [29]. When the hematoma volume exceeds 10 ml, the mortality comes close to 100% [30]. Our results confirmed that the mortality of patients with hematoma volume >10 ml was 71.4% (5/7), and the mortality of patients with hematoma volume of 5–10 ml was <8.3% (1/11). Univariate analysis showed a significant difference in the influence of hematoma volume and hematoma clearance rate on prognosis (*P* < 0.05), suggesting that hematoma volume and hematoma clearance rate may be factors affecting the prognosis of brainstem hemorrhage. Multivariate logistic regression analysis confirmed that the hematoma volume was an independent factor affecting the death of patients with brainstem hemorrhage (*P* < 0.05), while the hematoma volume was a risk factor (*P*: 0.048). The results were consistent with those previously reported in the literature [29]. Chung and Park classified brainstem hemorrhage into four types based on the characteristics of brain CT imaging, of which the survival rate of unilateral tegmental hemorrhage was 94.1%; however, the survival rates of basal tegmental type, bilateral tegmental type, and giant type were 26.1%, 14.3%, and 7.1%, respectively [29]. In the present study, we found that the survival rate of patients with unilateral tegmental brainstem hemorrhage was close to 100%, and the other types had higher mortality, which was consistent with the previous literature reports. Other studies argued that the GCS score could be used as an independent risk factor for SHBSH death [19]. In this study, two patients with preoperative GCS score ≥6 points survived, while the mortality rate of 17 patients with a preoperative GCS score of 3 to 5 points was 35.3% (6/17). Univariate analysis showed that the GCS score had no statistical significance for the prognosis of patients with brainstem hemorrhage (*P* = 1.000), which was inconsistent with previous literature reports, and may be related to the small sample size in this study. According to previous studies, about 39.5% of PBSH broke into the ventricular system [2], and the incidence of hydrocephalus was as high as 30.3% when the hemorrhage was located around the fourth ventricle and midbrain aqueduct, with a significantly increased risk of death and poor prognosis [22, 31]. We found that the presence of hydrocephalus was not associated with surgical results (*P* = 0.071), which was inconsistent with the literature and could be attributed to the small sample size. In addition, it was confirmed that men were significantly more affected by brainstem hemorrhage than women; however, we found no significant difference in gender and age, which was consistent with the literature [22].

This study has a few limitations. This is a single-center, retrospective study with a small sample size and short follow-up time. Further prospective multicenter studies are needed to confirm the long-term efficacy.

In conclusion, early microsurgical treatment can reduce mortality and improve the prognosis of SHBSH patients.

## Figures and Tables

**Figure 1:**
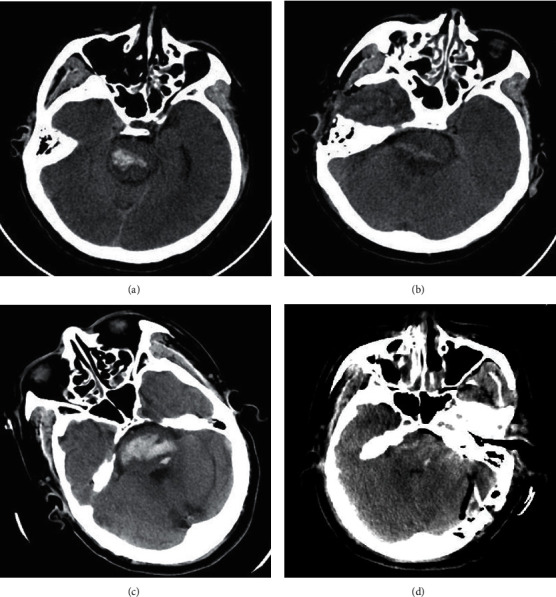
Forty-eight years old, male, who was admitted to the hospital with sudden unconsciousness for 3 hours. Physical examination: The patient was in coma with respiratory failure and tracheal intubation. Bilateral pupils were 1.5 mm, and the light reflex was negative. The patient was quadriplegic and bilateral Babinski's sign positive. Preoperative brain CT showed that the brainstem hematoma was located in the upper part of the pons, which was of unilateral tegmental type (a). The hematoma was cleared by subtemporal approach. Brain CT at 24 hours after operation showed that pons hemorrhage was cleared satisfactorily (b). Fifty-four years old, male, admitted to the hospital with sudden unconsciousness for 1 hour. Physical examination: The patient was in coma with bilateral miosis and negative for light reflex. The patient presented with quadriplegia, hypotonia, and bilateral Babinski's sign positive. Preoperative brain CT showed that the hematoma was located in the middle and lower part of the pons, which was of bilateral tegmental type (c). The hematoma was cleared through the retrosigmoid sinus approach. Brain CT at 24 hours after operation showed that pons hematoma was completely cleared and the fourth ventricle was clear (d).

**Table 1:** The modified Rankin scale (mRS).

Level	Details of the modified Rankin scale
0	No symptoms.
1	No significant disability. Able to carry out all usual activities, despite some symptoms.
2	Slight disability. Able to look after own affairs without assistance, but unable to carry out all previous activities.
3	Moderate disability. Requires some help, but able to walk unassisted.
4	Moderately severe disability. Unable to attend to own bodily needs without assistance, and unable to walk unassisted.
5	Severe disability. Requires constant nursing care and attention, bedridden, incontinent.
6	Dead.

**Table 2:** The 30-day and 90-day mortality in patients with brainstem hemorrhage.

	Number of cases (*n*)	Yes	No	Mortality rate (%)
30-Day mortality rate	*n* = 19	4	15	21.1
90-Day mortality rate	*n* = 19	6	13	31.6

**Table 3:** Univariate analysis of prognosis of patients with brainstem hemorrhage.

	Death (*n* = 6)	Survival (*n* = 13)	Mortality rate (%)	*P*
GCS score				1.000
3–5 points	6	11	35.3	
≥6 points	0	2	0.0	
Hematoma volume				0.010
5–10 ml	1	11	8.3	
≥10 ml	5	2	71.4	
Operation time				0.605
7–24 h	1	5	16.7	
≤6 h	5	8	38.5	
Combined hydrocephalus				0.071
Yes	3	1	75.0	
No	3	12	20.0	
Age	44.67 ± 8.38	51.54 ± 6.89	—	0.076
Gender				0.320
Male	3	10	23.1	
Female	3	3	50.0	
Hematoma clearance rate				0.041
≥80%	1	10	9.1	
<80%	5	3	62.5	
Hematoma type				0.511
Bilateral tegmental type	3	5	37.5	
Massive type	1	1	50.0	
Basal tegmental type	2	3	40.0	
Unilateral tegmental type	0	4	0.0	
Complicated with hydrocephalus				1.000
No	5	12	29.4	
Yes	1	1	50.0	
Complicated with intracranial infection				—
No	6	13	31.6	
Yes	0	0	—	
Complicated with intracranial hemorrhage				0.222
No	4	12	25.0	
Yes	2	1	66.7	
Complicated with patient vegetative survival				1.000
No	5	11	31.3	
Yes	1	2	33.3	

Two independent sample *t* tests were used to compare the difference in age between death and survival groups, and Fisher's exact test was used to compare other indicators.

**Table 4:** Multivariate logistic regression analysis for the prognosis of patients with brainstem hemorrhage.

	*B*	SE	Wald	df	*P*	OR	95% CI for OR	
							Lower	Upper
Hematoma volume (ml)	2.909	1.474	3.895	1	0.048	18.332	1.020	329.458
Hematoma clearance rate 24 h	−2.329	1.501	2.407	1	0.121	0.097	0.005	1.846
Constant	−1.139	1.210	0.886	1	0.346	0.320		

## Data Availability

The study data presented may be made available from the corresponding author upon reasonable request.
